# Does pharmacogenetic testing optimize antidepressant effectiveness in major depressive disorder? Data from a double-blind randomized controlled trial in a real-world clinical setting

**DOI:** 10.1192/j.eurpsy.2025.10132

**Published:** 2025-11-24

**Authors:** Alessandra Minelli, Stefano Barlati, Stefano Bignotti, Valentina Menesello, Gabriele Nibbio, Giulia Perusi, Alessia Muscarella, Lisa Buson, Ughetta Bosco Umbertino, Paolo Martini, Rosana Carvalho Silva, PANDORA Study Group, Edoardo Spina, Giovanni Battista Tura, Antonio Vita, Massimo Gennarelli

**Affiliations:** 1 Department of Molecular and Translational Medicine, University of Brescia, Brescia, Italy; 2 Genetics Unit, IRCCS Istituto Centro San Giovanni di Dio Fatebenefratelli, Brescia, Italy; 3 Department of Mental Health and Addiction Services, ASST Spedali Civili of Brescia, Brescia, Italy; 4 Department of Clinical and Experimental Sciences, University of Brescia, Brescia, Italy; 5 Psychiatry Unit, IRCCS Istituto Centro San Giovanni di Dio Fatebenefratelli, Brescia, Italy; 6 Clinical and Experimental Medicine, University of Messina, Messina, Italy

**Keywords:** antidepressant treatment, depression, genetics, pharmacogenetic testing, randomized controlled trial

## Abstract

**Background:**

Major depressive disorder (MDD) exhibits significant heterogeneity in treatment responses, necessitating multiple pharmacological trials to achieve therapeutic success. Pharmacogenetic (PGx) testing has emerged as a promising tool to personalize antidepressant (AD) treatments, though its clinical utility remains controversial.

**Methods:**

This study assessed the efficacy of PGx-guided treatment in improving clinical outcomes among 287 MDD patients within the PANDORA trial, a prospective randomized, participant- and rater-blinded, controlled trial conducted in Italy. A total of 268 adults with moderate-to-severe MDD were randomized into Treated as Usual (TAU) or Treated with Genetic Test Guide (TGTG). Patients were assessed using the Hamilton Depression Rating Scale (HAM-D17), Beck Depression Inventory II (BDI-II), Beck Anxiety Inventory, MINI-ICF-APP for psychosocial functioning, and the UKU Side Effects Rating Scale, at baseline and at 4, 8, and 12 weeks.

**Results:**

Both groups demonstrated significant symptom improvement over the 12-week period. No significant differences were observed between the groups in terms of response and remission rates, measured by HAM-D17 and BDI-II, at weeks 8 and 12. Notably, in the BDI-II symptom cluster analysis, significant differences were found only in neurovegetative symptoms, with TGTG patients showing greater improvement at the 4-week and 8-week follow-up visits. Among patients with severe baseline symptoms, those in the TGTG group exhibited greater symptom reduction and higher response rates at week 8.

**Conclusions:**

These findings suggest that while PGx testing did not significantly improve overall treatment efficacy in MDD compared to TAU, it may offer benefits in managing patients with severe symptoms and specific symptom domains.

## Introduction

Major depressive disorder (MDD) is the most common psychiatric illness, leading to functional impairment, reduced quality of life, and a substantial socioeconomic burden [1]. The disorder is characterized by a heterogeneous clinical presentation, encompassing a wide range of symptoms, severities, and trajectories, complicating diagnosis and treatment. Depression diagnosis and management challenge clinicians due to its heterogeneity, unpredictable course, and variable treatment response. Pharmacological treatment remains the first-line intervention for MDD patients and includes a wide range of antidepressants (AD). However, only about 30% respond adequately to the first AD treatment and achieve remission, while 15–30% develop treatment-resistant depression (TRD) [2, 3]. Many MDD patients discontinue AD treatment due to tolerability problems, such as side effects [4, 5]. Identifying the optimal treatment often requires multiple trials, with the likelihood of success diminishing with each unsuccessful attempt. This trial-and-error approach prolongs unremitted disease, worsens long-term prognosis, and adds medical, social, and economic burdens [6]. Consequently, there is a need for more precise, individualized strategies to improve outcomes and reduce the burden of MDD.

The efficacy and tolerability of AD treatment are complex and heterogeneous traits influenced by various biological and environmental factors. Difficulties in finding the optimal treatment in an individualized manner and achieving complete remission may be due to biological and environmental heterogeneity among patients with MDD [7, 8]. This suggests that biomarkers for AD response could assist clinicians in guiding treatment on an individual level. Pharmacogenetic (PGx) tests may improve treatment efficacy by predicting outcomes and reducing discontinuation due to side effects [9]. PGx testing employ genetic information for guide medication selection and dosing, aiming to enhance therapeutic outcomes and minimize adverse effects. By identifying genetic variations that affect drug metabolism and response, PGx tests can help tailor antidepressant therapy to individual patients, reducing the time to achieve remission and improving treatment success.

Studies have been performed to evaluate the utility of PGx tests in the treatment of MDD patients, including observational and randomized controlled trials (RCTs), with conflicting results. While PGx tests appear promising, their impact remains limited without meaningful advances in AD tolerability [10, 11]. Reviews, including systematic reviews and meta-analyses, suggest that PGx tests may improve response and remission rates and reduce adverse drug effects (ADEs), but findings remain inconsistent [10–14]. Trials with stratified analyses indicate PGx tests are more effective in patients with severe depressive symptoms and/or multiple unsuccessful medication attempts, showing higher response and remission rates [15, 16]. However, limitations such as heterogeneous study designs, blinding biases, varied trial durations and assessment time points, diverse patient populations, and uncertain cost-effectiveness continue to hinder evaluation and broader adoption of PGx tests in clinical practice [10]. Further research is needed to establish the clinical utility, cost-effectiveness, and best practices for PGx-guided treatment in MDD.

Given the potential utility of PGx tests in clinical settings and the heterogeneous and limited results in the literature regarding their practical implications, we conducted a RCT to evaluate the effects of PGx tests. This article presents the results of the PANDORA trial [17], an observational prospective randomized, participant- and rater-blinded, controlled trial, designed to evaluate the clinical efficacy of a combinatorial PGx test in guiding clinicians’ treatment decision making in a naturalistic setting among Italian MDD patients.

## Methods

### Study design

The PANDORA trial was a 12-week, observational prospective randomized, participant- and rater-blinded, controlled trial [17] registered at www.clinicaltrials.gov (NCT04615234), aimed to determine if pharmacogenetics could guide therapeutic decisions for MDD patients and improve treatment outcomes by maximizing drug efficacy. The study was approved by the Local Ethics Committees (CEIOC IRCCS Istituto Centro San Giovanni di Dio Fatebenefratelli, Brescia N: 43/2018, and Ethics Committee of ASST Spedali Civili of Brescia N: NP 3347) and performed in accordance with the Declaration of Helsinki. Patients provided written informed consent after receiving explanation of procedures and goals, following institutional guidelines.

The full study design is described elsewhere [17]. Patients were enrolled at screening between February 2020 and February 2024. Eligible patients were randomized 1:1 to Treated as Usual (TAU) or Treated with Genetic Test Guide (TGTG) (intervention). Pharmacogenomic testing was performed between screening and baseline visits. For TGTG patients, the PGx test report was released to clinicians for medication decisions. TAU patients were subjected to sample collection procedures identical to the experimental group, but PGx results were withheld, and clinicians were asked to manage controls according to the standard of care. All involved clinicians were asked to indicate, through a multiple-choice questionnaire, which factors they considered when deciding on drug therapy, including PGx results, clinical or pharmacological history, current psychopathological aspects, somatic or psychiatric comorbidities. Clearly, in the group of TAU prescribers, the possibility of choice, based on the results (green, yellow, red) of the pharmacogenetic report, was not indicated.

Patients were assessed for symptomatology using the Hamilton Depression Rating Scale (HAM-D17), Beck Depression Inventory II (BDI-II), Beck Anxiety Inventory (BAI), MINI-ICF-APP to monitor psychosocial functioning, and the UKU Side Effects Rating Scale, by trained raters at study initiation (baseline- T0) and at the 4 (T4), 8 (T8), and 12 (T12) weeks follow-up visits. Patients and raters were blinded to the patient’s assigned study group until the end of the trial. Medication changes in both groups (new medications, discontinuation, and dose adjustments) and prescription use were collected at all study visits, along with relevant information, including hospitalizations, medical visits, disability, and stressful events between visits.

### Study participants

**Figure 1.**
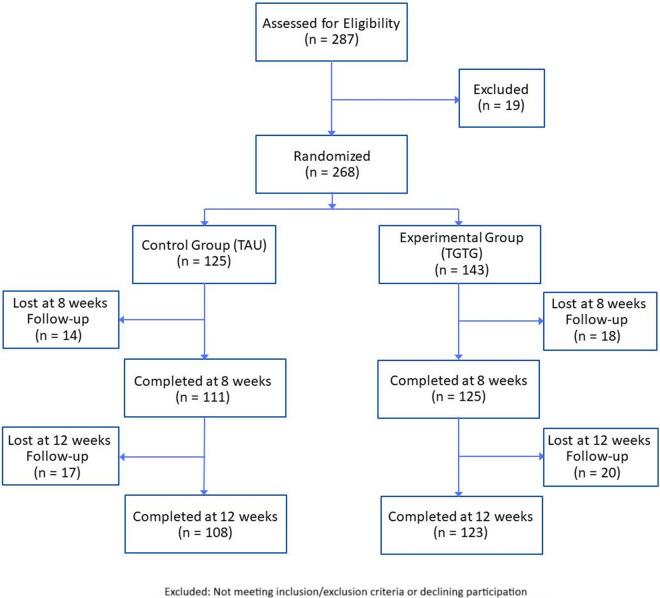
Flowchart of participant assessment, enrolment, randomization, exclusion, and follow-up.

A total of 287 patients was assessed for eligibility, with 19 excluded, resulting in 268 enrolled and randomized (Figure 1). Participants were Caucasian, aged 18–65, diagnosed with moderate or severe MDD (HAM-D17 score ≥14) according to DSM-5 criteria. Exclusion criteria included cognitive impairment (Mini-Mental State Examination, MMSE <24), neurological disorders, MDD with psychotic features, bipolar I and II disorders, schizophrenia spectrum and other psychotic disorders, obsessive–compulsive disorder, post-traumatic stress disorder, alcohol and substance abuse in the last 3 months, comorbid personality disorders (cluster A and/or B), pregnancy, and severe medical illnesses. Diagnoses were confirmed using the Italian version of the Structural Clinical Interview for DSM-5 disorders (SCID-5-CV) and the Structural Clinical Interview for personality disorders (SCID-5-PD). Patients were referred to Brescia’s psychiatric services (University Department of Mental Health, Spedali Civili Hospital and IRCCS Istituto Centro San Giovanni di Dio Fatebenefratelli – Brescia, Italy) for a new AD due to inadequate response (lack of improvement or intolerable side effects as reported by the patient or clinician).

### DNA extraction and pharmacogenetic testing

Details of the genetic testing methodology, including sample collection, DNA extraction, genotyping, and pharmacogenomic analysis, are provided in the following paragraphs. Buccal swabs were collected from all patients prior to randomization using FLOQSwab hDNA buccal brushes (Copan Brescia, Italy). Genomic DNA extraction was performed with a Quick DNA Miniprep plus Kit (ZymoResearch, CA, USA) according to the manufacturer’s instructions. Our PGx test included the following seven genes: *CYP2D6*, *CYP2C19*, *FKBP5*, *HTR1A*, *HTR2A*, *SLC6A4*, *MC4R.* We investigated the *CYP2D6*, *CYP2C19* genes because the PharmGKB database (www.pharmgkb.org) reportes moderate or high clinical annotation of evidence of their association with AD response and/or side effects in MDD patients. Moreover, we selected *FKBP5*, *HTR1A*, *HTR2A*, and *SLC6A4* due to their relevant roles as pharmacogenes. *MC4R* was selected for its key role in the development of weight gain side effect associated with amisulpride. Thirty single-nucleotide polymorphisms (SNPs) were genotyped with customized TaqMan OpenArray plates on a QuantStudio 12K Flex Real-Time PCR System (Applied Biosystems, Foster City, CA, USA) according to the manufacturer’s instructions, while the 5-HTTLPR (short/long allele) was genotyped by PCR amplification. Moreover, copy number variation (CNV) of the *CYP2D6* gene was evaluated using the TaqMan MGB probe chemistry (TaqMan Copy Number Assay, Thermo Fisher Scientific) specific for *CYP2D6* exon 9 and run on a StepOnePlus Real-Time PCR system (Applied Biosystems) according to the manufacturer’s instructions. The analysis was performed with CopyCaller Software (Applied Biosystems). Using AlleleTyper Software (Life Technologies, CA, USA), we integrated the SNP genotyping results with the copy number information for the *CYP2D6* gene to obtain all the *CYP2D6* and *CYP2C19* diplotypes. The translation was based on the translation table obtained from the PharmGKB database.

According to both the Clinical Pharmacogenetics Implementation Consortium (CPIC) and the Dutch Pharmacogenetics Working Group (DPWG) guidelines, the report categorized the ADs allowed for use in Italy into three recommended categories: (1) “use as directed” (labeled “green”), (2) “use with caution” (labeled “yellow”), and (3) “use with extreme caution” (labeled “red”) (see Supplementary Figure S1 for an example of PGx report). These warnings were accompanied by brief descriptions of the pharmacokinetics and/or pharmacodynamics reasons for caution (e.g., lack of efficacy or toxicity), along with recommendations for appropriate clinical action. The current AD was excluded from the report to avoid clinician bias in decision making. Before study initiation, training was provided to all participating investigators on the interpretation of genetic testing results and the relevance of each genetic variant to pharmacotherapy.

Refer to our protocol publication [17] and Supplementary Figure S1 for detailed information and a PGx report example.

### Outcomes

The primary outcome was the change in HAM-D17 scores from baseline to 8 weeks. Secondary outcomes included HAM-D17 symptom improvement at 12 weeks, response and remission rates at 8 and 12 weeks, and symptom improvement, response, and remission rates at 8 and 12 weeks according to the BDI-II. Tertiary outcomes included changes in anxiety symptoms at 8 and 12 weeks compared with baseline measured by the BAI, and changes in psychosocial functioning at 8 and 12 weeks compared with baseline measured by the MINI-ICF-APP. Safety and tolerability outcomes included side effects at 8 and 12 weeks assessed by the UKU Side Effect Rating Scale.

Response was defined as a ≥50% decrease in the assessment of interest (HAM-D17, BDI-II) at weeks 8 and 12 compared with the baseline. Remission was defined as a score ≤7 for HAM-D17 and ≤9 for BDI-II.

### Statistical analysis

Patient demographics and clinical characteristics were assessed using descriptive statistics. Analysis of variance (ANOVA) or chi-squared tests were used to analyze differences in continuous and categorical variables, respectively, among groups. Analyses were performed for patients completing the study through week 8. A mixed model for repeated measures (MMRM) examined time and group effects (TGTG vs. TAU) and their interaction for percentage changes from baseline in HAM-D17, BDI-II, BAI, and MINI-ICF-APP. A generalized linear mixed model was used for the UKU Side Effect Rating Scale and response/remission analyses. Dominant depression and anxiety factors were also considered using the HAM-D6 [18], the three-factor model of the BDI-II [19], and the two-factor model of the BAI [20]. All analyses were conducted using SPSS software package, version 21 (SPSS, Inc., USA).

## Results

### Participant characteristics

A total of 287 MDD patients were eligible, with 19 excluded for not meeting inclusion/exclusion criteria or declining participation (Figure 1). The remaining 268 were randomized to either the TGTG group (*n* = 143) or TAU group (*n* = 125) and followed for 12 weeks. The primary outcome analysis at 8 weeks included 236 patients (125 TGTG, 111 TAU), and 231 (123 TGTG, 108 TAU) completed the study (Figure 1). Participants had a mean age of 47 years, were predominantly female (72%), and had a baseline HAM-D17 score of 17.90. Most experienced moderate depressive episodes (64.9%), with no significant demographic or clinical differences between groups (Table 1).

**Table 1.** Sociodemographic and clinical features of the included MDD patients

	Total	TAU	TGTG	*p*
Sample size (*n*)	(268)	(125)	(143)	
Age in years, mean (SD)	46.50 (14.63)	46.93 (14.76)	46.12 (14.55)	0.652
Gender, *n* (%F)	192 (71.6)	89 (71.2)	103 (72.0)	0.881
Education in years, mean (SD)	12.59 (4.23)	12.26 (3.56)	12.89 (4.73)	0.223
Smokers, *n* (%)	84 (31.3)	40 (32.0)	44 (30.8)	0.828
Body mass index (BMI), mean (SD)	25.18 (5.36)	25.58 (5.62)	24.83 (5.12)	0.254
Age of onset in years, mean (SD)	31.18 (14.46)	31.52 (14.83)	30.87 (14.16)	0.715
Recurrent MDD, *n* (%)	224 (83.6)	101 (80.8)	123 (86.0)	0.250
Depression category				
Moderate (HAM-D17 14–18)	174 (64.9)	78 (62.4)	96 (67.1)	0.306
Severe (HAM-D17 19–22)	62 (23.1)	34 (27.2)	28 (19.6)	
Very Severe (HAM-D17 ≥23)	32 (11.9)	13 (10.4)	19 (13.3)	
Comorbidity with anxiety disorders, *n* (%)	85 (31.7)	43 (34,4)	42 (29.4)	0.377
Homotypic family history of psychiatric disorders, *n* (%)	127 (47.4)	58 (46.4)	69 (48.3)	0.762
Heterotypic family history of psychiatric disorders, *n* (%)	59 (22.0)	31 (24.8)	28 (19.6)	0.304
MMSE total score at the baseline, mean (SD)	28.98 (1.37)	28.94 (1.32)	29.01 (1.41)	0.677
HAM-D total score at the baseline, mean (SD)	17.90 (3.71)	17.93 (3.95)	17.88 (3.50)	0.918
BDI-II total score at the baseline, mean (SD)	28.38 (9.98)	29.36 (9.91)	27.53 (10.00)	0.135
BAI total score at the baseline, mean (SD)	23.36 (12.50)	23.90 (12.71)	22.84 (12.34)	0.488
MINI-ICF total score at the baseline, mean (SD)	8.46 (6.10)	8.54 (6.21)	8.39 (6.03)	0.847

Patients were referred due to inadequate response – defined as a lack of clinical improvement or intolerable side effects reported by the patient or clinician – primarily to selective serotonin reuptake inhibitors (SSRIs) (54.9%) (Supplementary Table S1). At baseline, SSRIs were the most prescribed psychotropic medications in both groups, accounting for half of all prescriptions (Supplementary Table S2).

### Symptom improvement, response, and remission for depression outcomes

**Figure 2.**
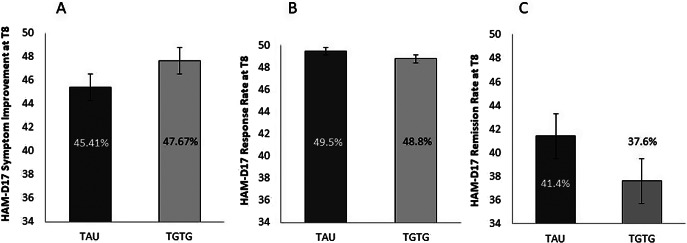
Percentage of symptom reduction (A), response (B), and remission (C) rates, as measured by the Hamilton Depression Rating Scale (HAM-D17) at T8, for the Treated with Genetic Test Guide (TGTG) and Treated as Usual (TAU) groups. No significant differences were observed between the groups.

For the primary outcome, analysis of HAM-D17 score indicated that the new AD significantly reduced depression symptomatology from baseline to week 8 (T0 = 18.03 ± 3.67; T8 = 9.67 ± 5.31; *F*_2,468_ = 425.64; *p* < 0.0001), whereas the arm group and the arm group by time point interaction were not significant (*F*_1,234_ = 0.81; *p* = 0.37; *F*_2,468_ = 0.93; *p* = 0.39, respectively). At T8, scores decreased by 47.7% in the TGTG group versus 45.4% in the TAU group (*p* = 0.52) (Figure 2A). No significant differences were observed in the secondary outcomes – response (TGTG = 48.8% vs. TAU = 49.5%; *p* = 0.91) (Figure 2B) and remission (TGTG = 37.6% vs. TAU = 41.4%; *p* = 0.55) (Figure 2C) – as evaluated by HAM-D17.

Consistent with HAM-D17 findings, BDI-II analysis showed symptom reduction with the new AD, as reported by patients, from baseline to T8 (T0 = 28.65 ± 10.18; T8 = 16.75 ± 10.22; *F*_2,466_ = 222.01; *p* < 0.0001), with no significant arm group by time point interaction (*F*_2,466_ = 0.45; *p* = 0.61). A slight difference between groups was noted (*F*_1,233_ = 4.04; *p* = 0.046), attributed to a significant difference at T4 (*p* = 0.038) and a trend at T8 (*p* = 0.084). At week 8, symptom reduction (TGTG = 42.2% vs. TAU = 38.9%; *p* = 0.45), response (TGTG = 46.4% vs. TAU = 37.3%; *p* = 0.16), and remission (TGTG = 31.2% vs. TAU = 24.5%; *p* = 0.26) did not differ significantly on BDI-II.

At 12 weeks, HAM-D17 analysis showed that the new AD reduced depression symptomatology from baseline to T12 (T0 = 18.06 ± 3.67; T12 = 7.85 ± 5.36; *F*_3,687_ = 431.47; *p* < 0.0001), while neither the arm group effect nor the arm group by time point interaction was significant (*F*_1,229_ = 0.91; *p* = 0.34; *F*_3,687_ = 0.45; *p* = 0.68, respectively). At T12, score reductions were 57.0% in TGTG versus 56.3% in TAU (*p* = 0.86), with no significant differences in response (TGTG = 65.0% vs. TAU = 65.7%; *p* = 0.91) or remission (TGTG = 53.7% vs. TAU = 53.7%; *p* = 0.96). Similar results were obtained for BDI-II at T12 (symptom reduction: T0 = 28.57 ± 10.14; T12 = 14.29 ± 11.72; *F*_3,687_ = 211.25; *p* < 0.0001; group effect *F*_1,229_ = 2.51; *p* = 0.11; arm group by time point interaction *F*_3,687_ = 1.82; *p* = 0.16). No significant differences were found in symptom reduction (TGTG = 48.5% vs. TAU = 51.2%; *p* = 0.59), response (TGTG = 56.9% vs. TAU = 59.3%; *p* = 0.72), or remission (TGTG = 50.4% vs. TAU = 39.8%; *p* = 0.11).

Analysis of the six most relevant HAM-D17 items revealed no significant effects at T8 or T12. The BDI-II’s three-factor model showed no significant effects for cognitive and affective symptoms at T8 and T12. However, somatic symptoms showed a significant group effect at T8 (*p* = 0.031) and a trend at T12 (*p* = 0.069). Post hoc comparisons revealed significant differences in scores between the groups at T4 (*p* = 0.030) and T8 (*p* = 0.028), with greater symptom improvement in TGTG.

### Symptom improvement, response, and remission for depression outcomes in severe depressed patients

Patients were stratified by baseline symptom severity by HAM-D17 median score (median = 17). The most significant results were observed for patients with severe symptoms (*n* = 134). The primary outcome analysis of HAM-D17 scores showed that the new AD significantly reduced depressive symptoms from baseline to T8 (T0 = 20.42 ± 3.16; T8 = 10.99 ± 5.61; *F*_2,264_ = 278.11; *p* < 0.0001). However, there were no significant effects for arm group or arm group by time point interaction (*F*_1,132_ = 2.87; *p* = 0.09; *F*_2,264_ = 3.14; *p* = 0.51). At T8, the TGTG group (*n* = 69) showed a 51.3% reduction versus 41.6% in TAU (*n* = 65), with a significant difference (*p* = 0.026). No significant group differences were observed for response and remission, assessed by HAM-D17. No significant effects were observed at T12. Analysis of the six most relevant HAM-D17 items revealed no statistically significant effects at either T8 or T12. However, at T8, there were trend-level effects noted for the group factor (*p* = 0.055) and for the difference in symptoms reduction between groups (*p* = 0.06).

**Figure 3.**
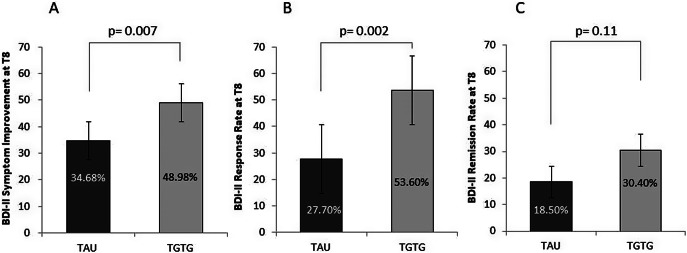
Percentage of symptom reduction (A), response (B), and remission (C) rates, as measured by the Beck Depression Inventory II (BDI-II) at T8, for severely depressed patients in the Treated with Genetic Test Guide (TGTG) and Treated as Usual (TAU) groups. Significant differences between groups were found for symptom reduction and response rates.

Analysis of the BDI-II scores revealed that the new AD intervention significantly reduced depression symptoms, as self-reported by the patients, from baseline to T8 (T0 = 32.04 ± 9.44; T8 = 18.49 ± 10.57; *F*_2,264_ = 155.14; *p* < 0.0001). The interaction between the arm group and time point was significant (*F*_2,264_ = 3.79; *p* = 0.029). The two groups showed a trend toward differences (*F*_1,132_ = 3.82; *p* = 0.053), with significant differences observed at T4 (*p* = 0.042) and T8 (*p* = 0.013). At T8, the TGTG group exhibited a greater symptom reduction compared to TAU (TGTG = 49.0% vs. TAU = 34.7%; *p* = 0.007) (Figure 3A), and a higher response rate (TGTG = 53.6% vs. TAU = 27.7%; *p* = 0.002) (Figure 3B). However, remission rates did not differ between groups (TGTG = 30.4% vs. TAU = 18.5%; *p* = 0.11) (Figure 3C). At T12, analysis showed a significant symptom reduction (*p* < 0.0001); however, neither the group effect nor the arm group by time point interaction reached significance (*p* = 0.11 and *p* = 0.08, respectively). No differences were observed between groups in symptom improvement (*p* = 0.47) or response (*p* = 0.39). However, remission rates showed a trend toward significance, favoring TGTG (TGTG = 47.8% vs. TAU = 32.3%; *p* = 0.07). When analyzing the BDI-II using the three-factor model, no significant effects were found for cognitive and affective symptoms at T8 and T12. In contrast, somatic symptoms showed significant reductions, as reported by patients, from baseline to T8 (*p* < 0.0001). The arm group by time point interaction and group effects were significant (*F*_2,264_ = 4.79; *p* = 0.011; *F*_1,132_ = 5.61; *p* = 0.019, respectively). Post hoc comparisons revealed significant differences between the two groups at T4 (*p* = 0.016) and T8 (*p* = 0.002), with the TGTG group showing greater improvement. Similar patterns were obtained at T12. At week 8, there was a significant difference in symptom reduction in TGTG versus TAU (46.9% vs. 30.4% respectively; *p* = 0.003).

### Symptom improvement for anxiety and psychosocial functioning outcomes

No significant differences were found for anxiety symptoms, measured by the BAI, or psychosocial functioning, assessed with the MINI-ICF-APP, at either T8 or T12. Analyses using the two-factor model of the BAI-II at both time points revealed no significant differences. Analyses of patients with more severe depressive symptomatology revealed trends toward significance for the somatic symptom cluster assessed by the two-factor model of the BAI-II (TGTG = 51.8% vs. TAU = 36.9%; *p* = 0.08) and for psychosocial functioning (TGTG = 32.3% vs. TAU = 7.7%; *p* = 0.07) at T8, indicating greater improvement in the TGTG group. However, these trends were not sustained in the analyses including T12 data.

### Adverse drug events

No significant differences in ADEs were found between groups at either T8 or T12, both in the overall sample and within the subgroup of patients with the most severe depressive symptoms. Additionally, the prescription of medications congruent or incongruent with the PGx report did not significantly influence the occurrence of ADEs.

### Medication congruency

Based on responses to the multiple-choice test, all psychiatrists in the TGTG group declared having thoroughly read the PGx report. Regardless of group assignment, the primary factors considered by psychiatrists when determining new AD treatments were clinical and symptomatologic evaluations, along with the patient’s pharmacological history. However, psychiatrists in the TGTG group were significantly influenced by the PGx test report, predominantly prescribing medications classified in the “green” category. In contrast, psychiatrists in the TAU group, who did not have access to the test results, relied only on their clinical expertise when prescribing medications (TGTG: green 65.5%, yellow 28.8%, red 5.8%; TAU: green 42.9%, yellow 42.9%, red 14.3%; *p* = 0.001).

Considering medication congruency or noncongruency, regardless of the group to which the patient was allocated, we found no significant effects on any measured outcomes at either T8 or T12.

## Discussion

This RCT investigated the clinical efficacy of a combinatorial PGx test in guiding clinicians’ treatment decision making among 268 MDD patients randomized into TGTG and TAU. Both groups experienced significant symptom reduction; however, no substantial differences were observed in the primary outcome – symptom change at 8 weeks measured by HAM-D17 – or in secondary outcomes, including symptom change at 12 weeks assessed by HAM-D17, and symptom improvement, response, and remission rates at 8 and 12 weeks according to BDI-II. Using the BDI-II three-factor model, a significant group effect for somatic symptoms was observed at T8, favoring TGTG. In stratified analyses of patients with severe baseline depression, the new AD significantly reduced symptoms over 8 weeks, with stronger improvements in the TGTG group, according to both HAM-D17 and BDI-II. While the TGTG group had higher response rates, remission differences were not significant. Symptom reductions persisted at 12 weeks, particularly for somatic symptoms, but improvements in cognitive and affective symptoms were not significant. Anxiety and psychosocial outcomes showed no significant differences, and adverse events were similar across groups. PGx-based medication congruency influenced prescribing patterns, with psychiatrists in the TGTG group predominantly prescribing “green” medications; however, it did not significantly impact outcomes.

Our findings align with existing literature. Observational studies and RCTs have explored PGx tests in MDD treatment, yielding mixed results, as effectiveness remains limited without advancements in AD tolerability [10, 11]. Many RCTs found no significant effect of PGx test on symptom improvements [15, 21–24] or side effects [21–23]. Different from our results, some RCTs reported better tolerability and fewer side effects in the PGx-guided groups [16, 25]. A recent review updated prior guidelines and found limited evidence supporting PGx-guided treatment in MDD, emphasizing that none of the reviewed studies were fully blinded and that efficacy results varied [12]. Another umbrella review and meta-analysis suggested that PGx-guided prescribing improves remission and response in MDD, though effects varied [13]. A systematic review, however, found no significant impact on outcomes from PGx-guided prescribing, suggesting a limited role for routine testing [14].

Our findings also align with studies analyzing patients with severe symptoms. Stratified analyses of this group often show significant differences with PGx testing [15, 16]. In patients with multiple unsuccessful medication attempts, possibly TRD patients, PGx groups showed higher response and remission rates, with the largest effect sizes in those with severe depression. No effects were observed for drug-naïve patients. This suggests PGx tests predominantly helped difficult-to-treat patients whose treatment resistance may relate to genetic background. PGx tests may be useful for patients with severe MDD, maximizing benefits and minimizing costs of pharmacogenomics analyses, which would be justified if the traditional treatment fails.

While findings suggest no substantial differences between TGTG and TAU in symptom alleviation, improvements in subgroups with more severe symptoms raise questions about the clinical significance of these results. Beyond statistical significance, it is crucial to explore whether these subgroup differences translate into improvements in treatment decisions and patient outcomes. Additionally, the absence of a significant effect highlights the need to consider potential limitations of PGx testing, such as its restricted gene panel and the complex interplay between genetic and environmental factors. Further discussion on the role of pharmacogenetics in personalizing antidepressant therapy, particularly in subpopulations with higher treatment resistance, may clarify its value in clinical practice.

This study presents several strengths. First, its randomized, controlled, participant- and rater-blinded design ensures an unbiased evaluation of PGx effects on treatment outcomes. A 12-week longitudinal assessment provides a better understanding of PGx testing effects, whereas most studies lasted only 8 weeks [21, 23, 25]. Second, the large sample size and diverse patient population improve the generalizability of the findings. Third, the study evaluates both clinical efficacy and tolerability, providing a comprehensive AD treatment analysis. Fourth, it employs a naturalistic setting, reflecting real-world clinical practice, and incorporates detailed longitudinal assessments using validated scales for depression, anxiety, psychosocial functioning, and side effects. Finally, our study addresses critical gaps in the literature by stratifying analyses based on patient severity and treatment history, identifying subgroups that benefit most from PGx guidance.

Our study also has limitations. Consistent with other studies, our PGx test includes a limited number of genes, restricting analyses to a few variants despite the complex pathophysiology of MDD. MDD response is also a complex phenotype involving many genes; no single gene or limited gene sets, even those for drug metabolism and targets, account for more than a fraction of illness risk or treatment course. Moreover, environmental factors, age, sex, and diet, often outweigh inherited determinants of drug metabolism and response. Despite this complexity, most PGx tests analyze selected polymorphisms within a few genes affecting the response to most antidepressants, mainly focusing on pharmacokinetic genes such as CYP2D6 and CYP2C19, while pharmacodynamic genes contribute only minimally. As a result, the interpretation of test outcomes is often not clear. This reduces the ability to comprehensively evaluate genetic factors influencing drug response, as many relevant genes and variants are excluded. While this may be a limitation, it is also an inherent constraint in most research utilizing available PGx tests. Thus, the insights provided by PGx tests are limited, hiding genetic contributors to medication efficacy, tolerability, or side effects that could enhance personalized treatments. In practice, among the included pharmacokinetic and pharmacodynamic genes, only two involved in drug metabolism, CYP2D6 and CYP2C19, significantly influence test results, while pharmacodynamic genes contribute only minimally to medication classification. Overall, these constraints highlight that current PGx tests provide only a partial view of the genetic architecture of antidepressant response, focusing on a limited set of genes and disregarding other relevant factors that influence treatment response. To address this imbalance, we developed an informative report that distinguishes pharmacokinetic from pharmacodynamic effects, enabling clinicians to understand the specific genetic factors influencing each aspect of treatment recommendations. Another limitation is the concomitant use of non-psychotropic medications, which could affect drug metabolism and influence results. Our findings should be interpreted in the context of limitations similar to those reported in other RCTs, including study design, trial duration, outcome assessments, patient groups studied, and cost-effectiveness, that seem to restrict the evaluation of the utility of PGx tests and their clinical adoption [10]. These limitations should be addressed in future studies to enable generalization of results and their clinical application.

Pharmacogenetic testing holds promise for improving depression treatment by tailoring AD therapy to individual genetic profiles, increasing treatment success, reducing side effects, and improving patient satisfaction. However, challenges related to cost, limited clinical evidence, and the complexity of psychiatric disorders must be addressed before PGx testing can become routine in clinical practice. Indeed, evidence for large-scale use of PGx tests is limited, and current data do not support their routine use for all patients with depression. Nevertheless, our findings highlight their potential benefit for a specific subgroup of patients, those presenting severe depressive symptoms, who has a higher probability to experience treatment failure. PG testing may be particularly useful for those carrying functional variants that increase the risk of treatment resistance or adverse drug effects. In these cases, PGx tests could guide a precision medicine approach, improving outcomes while reducing unnecessary costs. This is especially relevant because these patients often consume more healthcare resources. Future studies should evaluate this targeted use of PGx testing. Ongoing research, education, and collaboration will be key to realizing the full potential of PGx testing in psychiatry.

## Supporting information

10.1192/j.eurpsy.2025.10132.sm001 Minelli et al. supplementary material 1 Minelli et al. supplementary material

10.1192/j.eurpsy.2025.10132.sm002 Minelli et al. supplementary material 2 Minelli et al. supplementary material

## Data Availability

Data from the current study can be made available upon request to the corresponding author.

## Supplementary material

The supplementary material for this article can be found at http://doi.org/10.1192/j.eurpsy.2025.10132.
