# Supplementary material for: Does pharmacogenetic testing optimize antidepressant effectiveness in major depressive disorder? Data from a double-blind randomized controlled trial in a real-world clinical setting
Source: Eur Psychiatry. 2025 Nov 24;68(1):e175. doi: 10.1192/j.eurpsy.2025.10132 (PMC12780592; doi:10.1192/j.eurpsy.2025.10132)
Supplement: Minelli et al. supplementary material 1 — Minelli et al. supplementary material [file S0924933825101326sup001.pdf]

Report for patient XX_XX_XX

Drug	Drug	Drug
Amitriptyline	Citalopram	Agomelatine
Clomipramine	Fluoxetine	Bupropion
Imipramine	Fluvoxamine	Duloxetine
Nortriptyline	Mirtazapine	Escitalopram
Trimipramine	Paroxetine	Mianserine
Venlafaxine		Reboxetine
		Sertraline
		Trazodone
		Vortioxetine
		Amisulpride

Amitriptyline

[FC] Reduced clinical efficacy and increased likelihood of side effects. Reduce initial dose by 25% compared to recommended.

Clomipramine

[FC] Reduced clinical efficacy and increased likelihood of side effects. Reduce initial dose by 25-30% compared to recommended.

Imipramine

[FC] Reduced clinical efficacy and increased likelihood of side effects. Reduce initial dose by 25-30% compared to recommended.

Nortriptyline

[FC] Reduced clinical efficacy and increased likelihood of side effects. Reduce initial dose by 25-40% compared to recommended.

Trimipramine

[FC] Increased likelihood of side effects. Reduce initial dose by 25% compared to recommended.

Venlafaxine

[FC] Reduced clinical efficacy and increased likelihood of side effects. An alternative molecule is recommended.

[FD] Possible reduction in clinical efficacy

Citalopram

[FD] Possible reduction in clinical efficacy

Fluoxetine

[FD] Possible reduction in clinical efficacy

Fluvoxamine

[FC] Possible presence of side effects, not clinically relevant. Start therapy at the recommended therapeutic dose.

Mirtazapine

[FC] No adverse effects in terms of efficacy or side effects. Start therapy at the recommended therapeutic dose.

[FD] Possible reduction in clinical efficacy

Paroxetine

[FC] Possible presence of side effects, not clinically relevant. Start therapy at the recommended therapeutic dose.

[FD] Possible reduction in clinical efficacy

Agomelatine

No data available.

Bupropion

No data available.

Duloxetine

No data available.

Escitalopram

No data available.

Mianserine

No data available.

Reboxetine

No data available.

Sertraline

No data available.

Trazodone

No data available.

Vortioxetine

No data available.

Amisulpride

No data available

Note: FC= Pharmacokinetics; FD= Pharmacodynamics
