# Supplementary material for: Does pharmacogenetic testing optimize antidepressant effectiveness in major depressive disorder? Data from a double-blind randomized controlled trial in a real-world clinical setting
Source: Eur Psychiatry. 2025 Nov 24;68(1):e175. doi: 10.1192/j.eurpsy.2025.10132 (PMC12780592; doi:10.1192/j.eurpsy.2025.10132)
Supplement: Minelli et al. supplementary material 2 — Minelli et al. supplementary material [file S0924933825101326sup002.docx]

**Supplementary Material**

**Supplementary Table 1**

Drug changed due to an inadequate response (lack of clinical improvement or intolerable side-effects as reported by the patient or treating clinician)

| Drug (CLASS) | TOT (n=268) | TAU (n=125) | TGTG (n=143) | p-value |
| --- | --- | --- | --- | --- |
| Vortioxetine (SMS*), n (%) | 38 (14.2) | 17 (13.6) | 21 (14.7) | .799 |
| Fluoxetine (SSRI), n (%) | 16 (6.0) | 9 (7.2) | 7 (4.9) | .427 |
| Paroxetine (SSRI), n (%) | 37 (13.8) | 16 (12.8) | 21 (14.7) | .655 |
| Sertraline (SSRI), n (%) | 39 (14.6) | 20 (16.0) | 19 (13.3) | .530 |
| Citalopram (SSRI), n (%) | 29 (10.8) | 8 (6.4) | 21 (14.7) | **.029** |
| Escitalopram (SSRI), n (%) | 25 (9.3) | 13 (10.4) | 12 (8.4) | .573 |
| Fluvoxamine (SSRI), n (%) | 1 (0.4) | 1 (0.8) | 0 (0.0) | .949 |
| Duloxetine (SNRI), n (%) | 23 (8.6) | 11 (8.8) | 12 (8.4) | .905 |
| Venlafaxine (SNRI), n (%) | 29 (10.8) | 15 (12.0) | 14 (9.8) | .561 |
| Amitriptyline (TCA), n (%) | 4 (1.5) | 2 (1.6) | 2 (1.4) | .892 |
| Clomipramine (TCA), n (%) | 2 (0.7) | 1 (0.8) | 1 (0.7) | .924 |
| Doxepin (TCA), n (%) | 1 (0.4) | 0 (0.0) | 1 (0.7) | >.999 |
| Trazodone (Atypical), n (%) | 5 (1.9) | 3 (2.4) | 2 (1.4) | .546 |
| Amisulpride (Atypical), n (%) | 2 (0.7) | 0 (0.0) | 2 (1.4) | >.999 |
| Bupropion (Atypical), n (%) | 7 (2.6) | 4 (3.2) | 3 (2.1) | .573 |
| Mirtazapine (Others), n (%) | 6 (2.2) | 2 (1.6) | 4 (2.8) | .509 |
| Others, n (%) | 4 (1.5) | 3 (2.4) | 1 (0.7) | .252 |
|  | | | | |
| (CLASS) | TOT (n=268) | TAU (n=125) | TGTG (n=143) | p-value |
| SSRI*, n (%) | 147 (54.9) | 67 (53.6) | 80 (55.9) | .700 |
| SNRI*, n (%) | 52 (19.4) | 26 (20.8) | 26 (18.2) | .589 |
| TCA*, n (%) | 7 (2.6) | 3 (2.4) | 4 (2.8) | .839 |
| OTHERS, n (%) | 62 (23.1) | 29 (23.2) | 33 (23.1) | .981 |

*SMS: Serotonin Modulator and Stimulator

*SSRI: Selective Serotonin Reuptake Inhibitors

*SNRI: Serotonin and Norepinephrine Reuptake Inhibitors

*TCA: Tricyclic Antidepressants

**Supplementary Table 2**

List of drugs prescribed in the trial

| NEW AD (CLASS) | TOT (n=268) | TAU (n=125) | TGTG (n=143) | p-value |
| --- | --- | --- | --- | --- |
| Vortioxetine (SMS*), n (%) | 20 (7.5) | 10 (8.0) | 10 (7.0) | .754 |
| Fluoxetine (SSRI), n (%) | 17 (6.3) | 9 (7.2) | 8 (5.6) | .591 |
| Paroxetine (SSRI), n (%) | 18 (6.7) | 15 (12.0) | 3 (2.1) | **.001** |
| Sertraline (SSRI), n (%) | 49 (18.3) | 18 (14.4) | 31 (21.7) | .124 |
| Citalopram (SSRI), n (%) | 5 (1.9) | 3 (2.4) | 2 (1.4) | .546 |
| Escitalopram (SSRI), n (%) | 28 (10.4) | 14 (11.2) | 14 (9.8) | .707 |
| Fluvoxamine (SSRI), n (%) | 5 (1.9) | 1 (0.8) | 4 (2.8) | .228 |
| Duloxetine (SNRI), n (%) | 58 (21.6) | 20 (16.0) | 38 (26.6) | **.036** |
| Venlafaxine (SNRI), n (%) | 38 (14.2) | 22 (17.6) | 16 (11.2) | .133 |
| Desvenlafaxine (SNRI), n (%) | 1 (0.4) | 1 (0.8) | 0 (0.0) | 0.949 |
| Amitriptyline (TCA), n (%) | 2 (0.7) | 2 (1.6) | 0 (0.0) | 0.426 |
| Clomipramine (TCA), n (%) | 1 (0.4) | 0 (0.0) | 1 (0.7) | >.999 |
| Nortriptyline TCA), n (%) | 3 (1.1) | 0 (0.0) | 3 (2.1) | 0.303 |
| Trazodone (Atypical), n (%) | 6 (2.2) | 3 (2.4) | 3 (2.1) | .868 |
| Amisulpride (Atypical), n (%) | 1 (0.4) | 0 (0.0) | 1 (0.7) | >.999 |
| Bupropion (Atypical), n (%) | 8 (3.0) | 3 (2.4) | 5 (3.5) | .599 |
| Mirtazapine (Others), n (%) | 3 (1.1) | 3 (2.4) | 0 (0.0) | 0.208 |
| No drug (drop out), n (%) | 5 (1.9) | 1 (0.8) | 4 (2.8) | .228 |
|  | | | | |
| (CLASS) | TOT (n=268) | TAU (n=125) | TGTG (n=143) | p-value |
| SSRI*, n (%) | 122 (45.5) | 60 (48.0) | 62 (43.4) | .446 |
| SNRI*, n (%) | 97 (36.2) | 43 (34.4) | 54 (37.8) | .568 |
| TCA*, n (%) | 6 (2.2) | 2 (1.6) | 4 (2.8) | .509 |
| OTHERS, n (%) | 38 (14.2) | 19 (15.2) | 19 (13.3) | .654 |
| No drug (drop out), n (%) | 5 (1.9) | 1 (0.8) | 4 (2.8) | .228 |

*SMS: Serotonin Modulator and Stimulator

*SSRI: Selective Serotonin Reuptake Inhibitors

*SNRI: Serotonin and Norepinephrine Reuptake Inhibitors

*TCA: Tricyclic Antidepressants
